# Supplementary material for: Multi-omics profiling reveals microenvironmental remodeling as a key driver of house dust mite-induced lung cancer progression
Source: bioRxiv. 2025 Sep 27:2025.09.24.678347. Preprint. [Version 1] doi: 10.1101/2025.09.24.678347 (PMC12485783; doi:10.1101/2025.09.24.678347)
Supplement: Supplement 1 [file NIHPP2025.09.24.678347v1-supplement-1.pdf]

bioRxiv preprint doi: https://doi.org/10.1101/2025.09.24.678347; this version posted September 29, 2025. The copyright holder for this preprint (which was not certified by peer review) is the author/funder, who has granted bioRxiv a license to display the preprint in perpetuity. It is made available under aCC-BY 4.0 International license.

Supplemental information

Multi-omics profiling reveals microenvironmental remodeling as a key driver of house dust mite-induced lung cancer progression

Shams Al-Azzam, Isabella Stuewe, Sunandini Sharma, Miki Yamada-Hara, Arisachi Tanaka, Kegan Stringer, Merna Behnam, Norah Al-Azzam, Shuvro Nandi, Maria Zhivagui, Janelle Duong, Ting Yang, Scott Herdman, Maripat Corr, Nicholas J.G Webster, Eyal Raz, Ludmil B Alexandrov, and Samuel Bertin

bioRxiv preprint doi: https://doi.org/10.1101/2025.09.24.678347; this version posted September 29, 2025. The copyright holder for this preprint (which was not certified by peer review) is the author/funder, who has granted bioRxiv a license to display the preprint in perpetuity. It is made available under aCC-BY 4.0 International license.

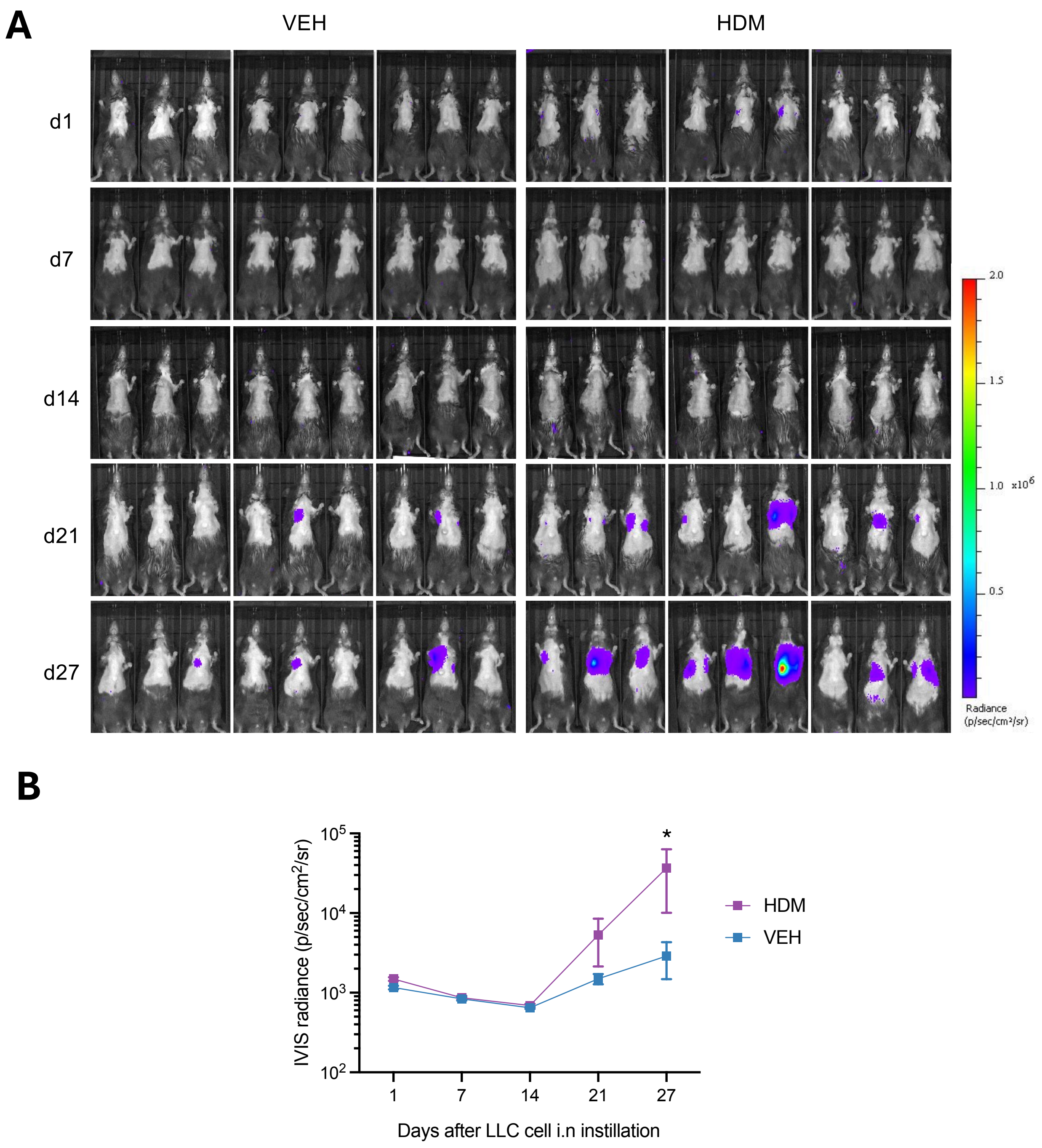

Figure S1. Kinetic of Tumor Growth in the LLC Orthotopic Lung Carcinoma Mouse Model. A) Representative IVIS images of tumor signals quantified and **B)** IVIS radiance in C57BL/6J WT mice (n = 9 mice/group) treated as shown in Figure 1A. Data are presented as mean ± SEM. Statistical significance was assessed by two-way ANOVA with post hoc Bonferroni's test; * p <0.05.

bioRxiv preprint doi: https://doi.org/10.1101/2025.09.24.678347; this version posted September 29, 2025. The copyright holder for this preprint (which was not certified by peer review) is the author/funder, who has granted bioRxiv a license to display the preprint in perpetuity. It is made available under aCC-BY 4.0 International license.

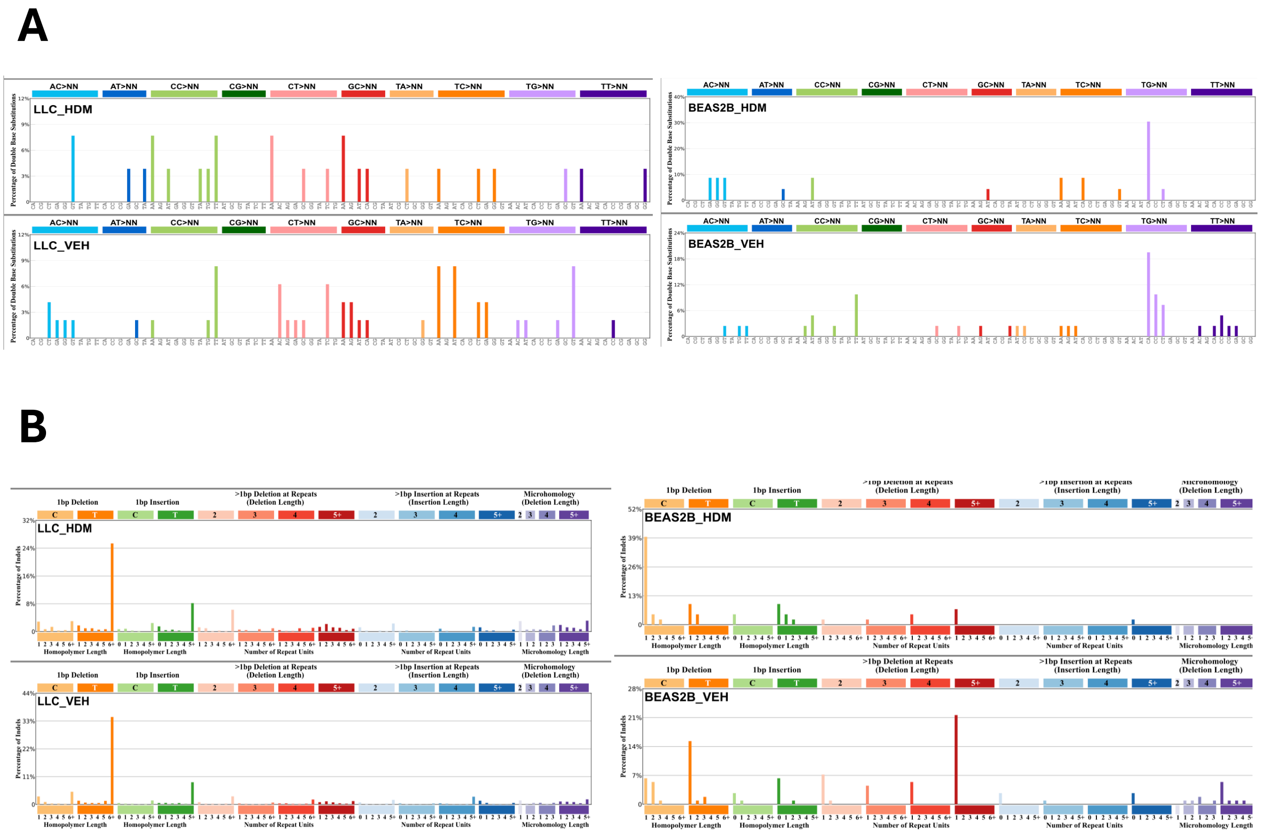

Figure S2. DBS78 and ID83 mutational profiles in HDM- and vehicle-treated LLC and BEAS-2B cells. A) Double-base substitution signatures (DBS78) for HDM- and VEH-treated LLC and BEAS-2B cell lines. **B)** Insertion-deletion signatures (ID83) for the same samples and conditions as in A.

bioRxiv preprint doi: https://doi.org/10.1101/2025.09.24.678347; this version posted September 29, 2025. The copyright holder for this preprint (which was not certified by peer review) is the author/funder, who has granted bioRxiv a license to display the preprint in perpetuity. It is made available under aCC-BY 4.0 International license.

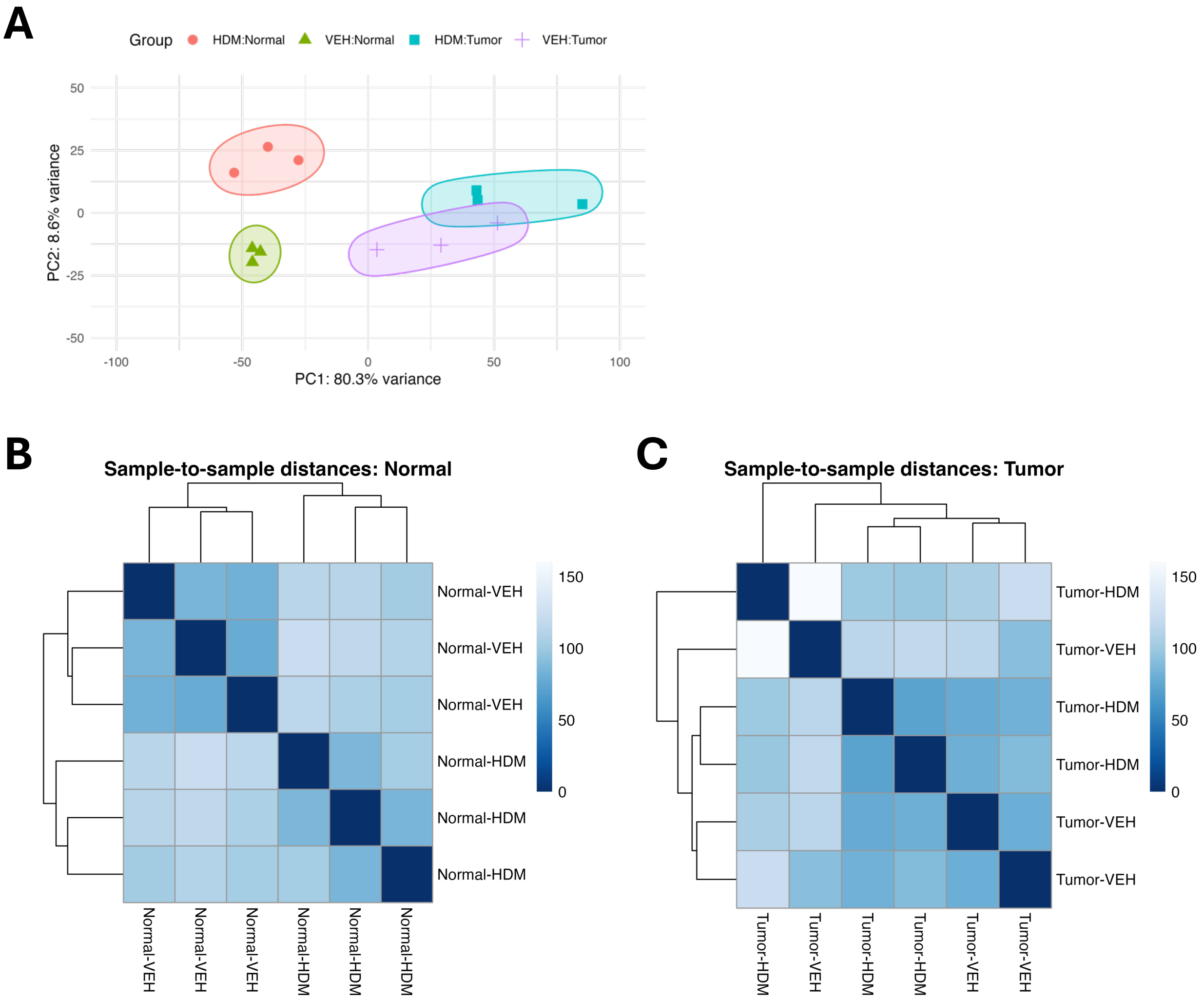

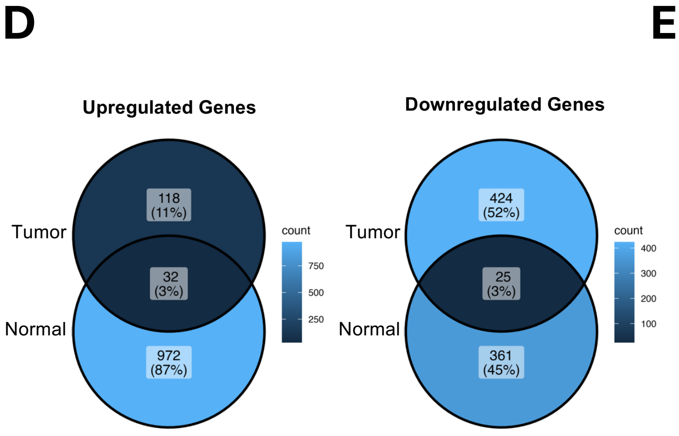

E

Common Upregulated Genes	Common Downregulated Genes
Angptl4	Rec8
Has1	Gata3
Hmox1	Cabcoco1
Per1	Tspan13
Mctp1	Ripply3
Il33	Tmem47
Alox5	Egfl7
Mrc1	Prom2
Gpcpd1	Ccna1
Cxcl1	Ces1f
Fam110c	1700012B09Rik
Saa3	Cyp1a1
Scamp5	Tnxb
Nxpe5	Smad6
Gda	Azgp1
Serpinb2	Tnfsf10
Igkv15-103	Colq
Ighg1	mt-Rnr1
Ighv1-64	mt-Rnr2
Igkv1-117	mt-Nd2
Igkv10-96	mt-Nd3
Igkv5-43	mt-Nd4
Ighv1-82	Rtl8a
Igkv14-111	Gm12216
Ighv1-72	Gm48084
Igkv12-44	
Ighv9-3	
Ighv2-2	
Ighv9-1	
Lilrb4b	
Gm30489	
Gm56730	

bioRxiv preprint doi: https://doi.org/10.1101/2025.09.24.678347; this version posted September 29, 2025. The copyright holder for this preprint (which was not certified by peer review) is the author/funder, who has granted bioRxiv a license to display the preprint in perpetuity. It is made available under aCC-BY 4.0 International license.

Figure S3. Sample Clustering in the RNA-seq Experiment. A) Principal component analysis (PCA) of samples. PCA was performed on log2(TPM+1) expression values (genes centered and scaled). Points are individual samples; color denotes exposure (HDM vs VEH) and shape denotes tissue (Normal vs Tumor). Ellipses show covariance-based group dispersion for visualization only (no hypothesis testing). **B)** Sample-to-sample distance heatmap of normal lung tissue, and **C)** of tumor samples. Pairwise Euclidean distances were computed from log2(TPM+1) expression and clustered by hierarchical clustering. The top dendrograms show clustering of samples; the left dendrograms show clustering of the distance profiles. Darker blue indicates smaller distances (greater similarity); lighter shades indicate larger distances (lower similarity). **D)** Venn diagrams showing overlap of upregulated (left) and downregulated (right) differentially expressed genes (DEGs) between normal and tumor tissues. Numbers indicate unique and shared gene counts, along with their relative percentages. **E)** Table listing DEGs shared between normal lung tissue and tumors, with upregulated genes on the left and downregulated genes on the right. Differential expression was assessed with a negative-binomial model (DESeq2, Wald test) with multiple testing controlled by Benjamini-Hochberg false discovery rate (FDR); genes with FDR < 0.05 were considered significant.

bioRxiv preprint doi: https://doi.org/10.1101/2025.09.24.678347; this version posted September 29, 2025. The copyright holder for this preprint (which was not certified by peer review) is the author/funder, who has granted bioRxiv a license to display the preprint in perpetuity. It is made available under aCC-BY 4.0 International license.

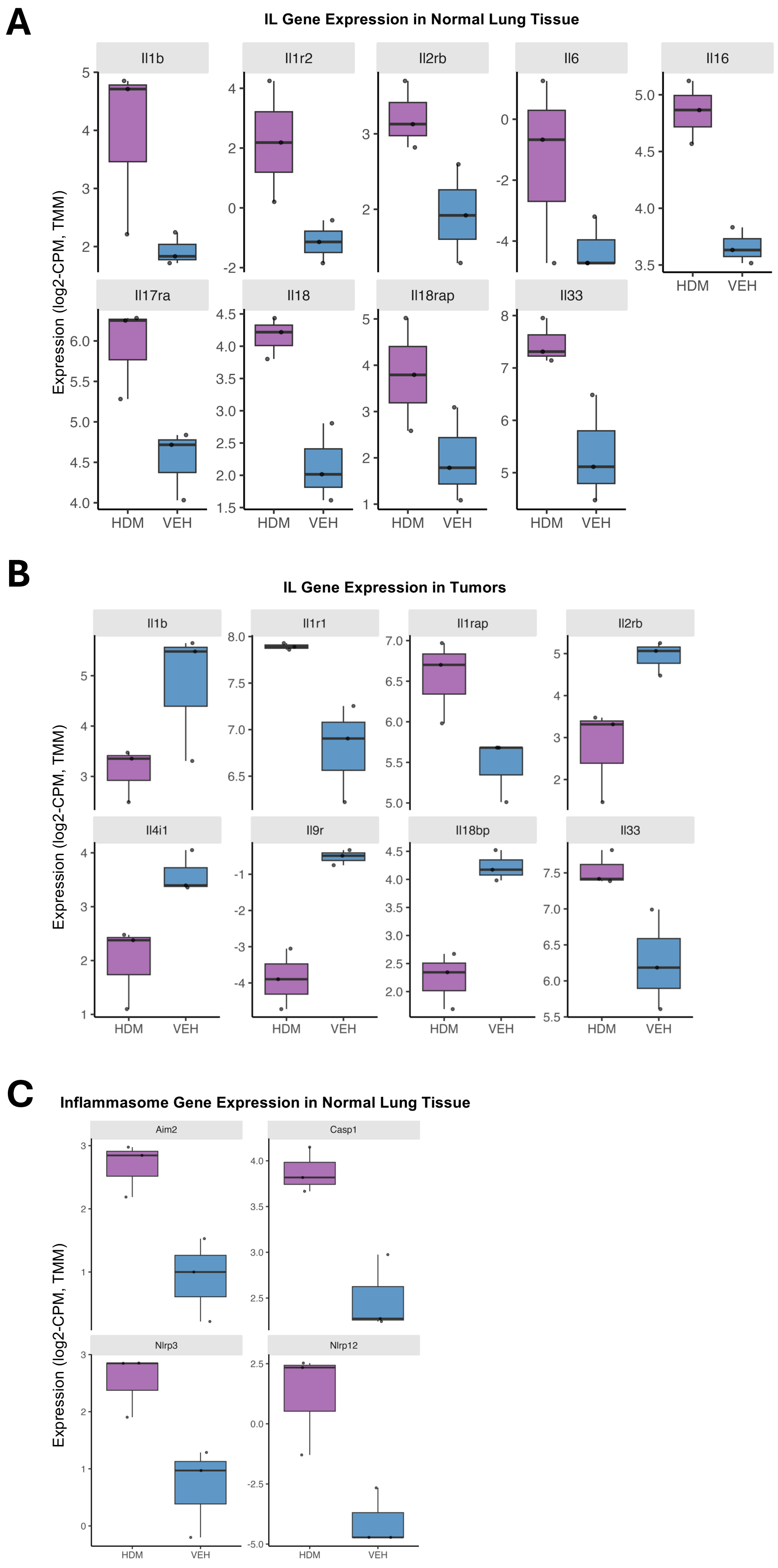

bioRxiv preprint doi: https://doi.org/10.1101/2025.09.24.678347; this version posted September 29, 2025. The copyright holder for this preprint (which was not certified by peer review) is the author/funder, who has granted bioRxiv a license to display the preprint in perpetuity. It is made available under aCC-BY 4.0 International license.

Figure S4. HDM Exposure Induces Distinct Immune Gene Signatures in Normal Lung Tissues and Tumors. A) Boxplots showing trimmed mean of M values (TMM)-normalized expression (log2-counts per million [CPM]) for selected interleukin (IL) cytokines and receptors in mice treated with HDM (purple) or VEH (blue) in normal lung tissue, and **B)** in tumors. Boxes indicate the median and interquartile range (IQR); whiskers extend to 1.5× the IQR; points are individual samples. **C)** Boxplots showing TMM-normalized expression (log2-CPM) for selected inflammasome-related genes in mice treated with HDM (purple) or VEH (blue) in normal lung tissue. Genes displayed were pre-selected as differentially expressed in the global analysis (DESeq2 Wald test with Benjamini-Hochberg FDR < 0.05).

bioRxiv preprint doi: https://doi.org/10.1101/2025.09.24.678347; this version posted September 29, 2025. The copyright holder for this preprint (which was not certified by peer review) is the author/funder, who has granted bioRxiv a license to display the preprint in perpetuity. It is made available under aCC-BY 4.0 International license.

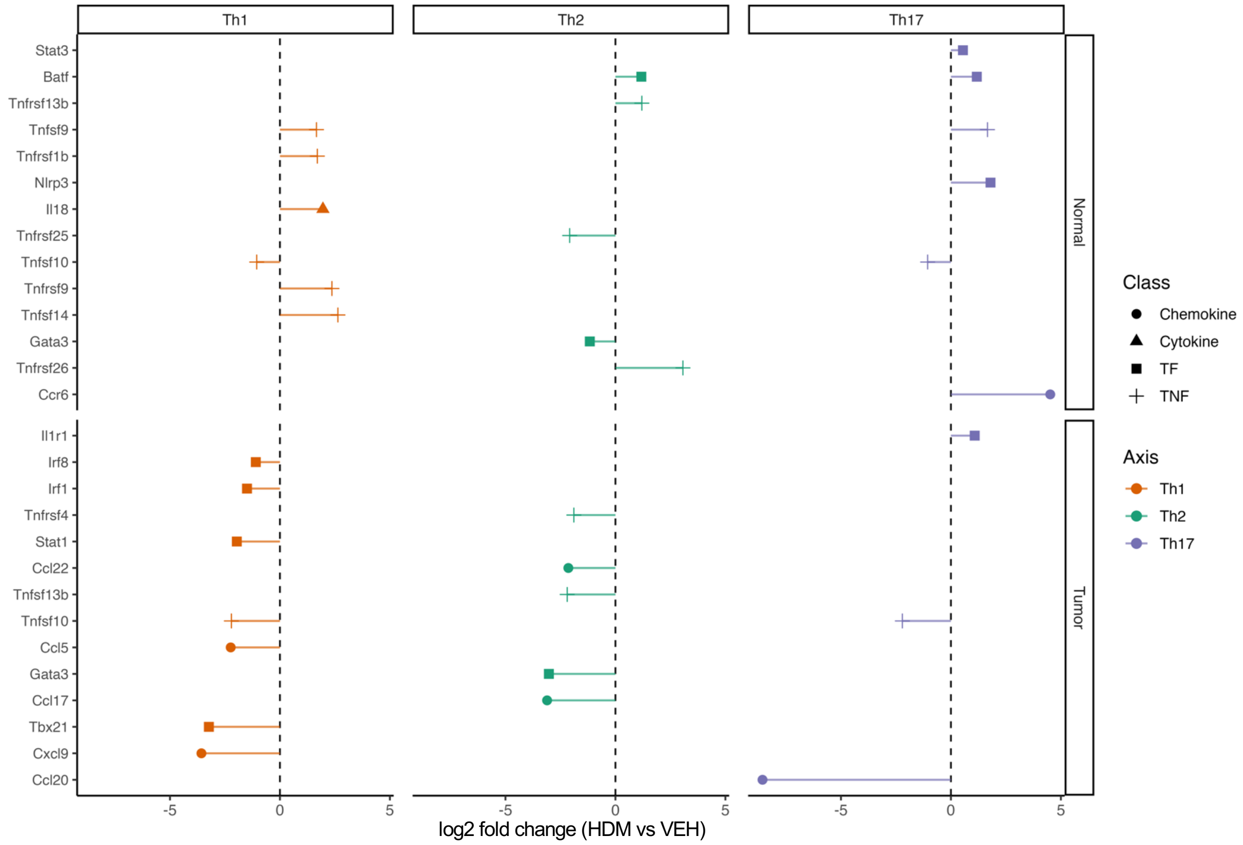

Figure S5. HDM Exposure Enhances T Cell Responses in Normal Lung Tissues but Suppressed Them in Tumors. Forest plots showing the log2 fold change (HDM vs VEH) for curated Th1, Th2, and Th17-associated genes in normal lung tissue (top) and tumors (bottom). Points represent the estimated log2 fold change for each gene. Point color encodes Th axis, and point shape denotes gene class: chemokine, cytokine, transcription factor (TF), or TNF family members. Differential expression was assessed per gene using a DESeq2 negative-binomial model with the Wald test (two-sided) on size-factor-normalized counts. *p* values were adjusted by Benjamini-Hochberg FDR in the global analysis; only genes with nominal $p < 0.05$ are shown.

bioRxiv preprint doi: https://doi.org/10.1101/2025.09.24.678347; this version posted September 29, 2025. The copyright holder for this preprint (which was not certified by peer review) is the author/funder, who has granted bioRxiv a license to display the preprint in perpetuity. It is made available under aCC-BY 4.0 International license.

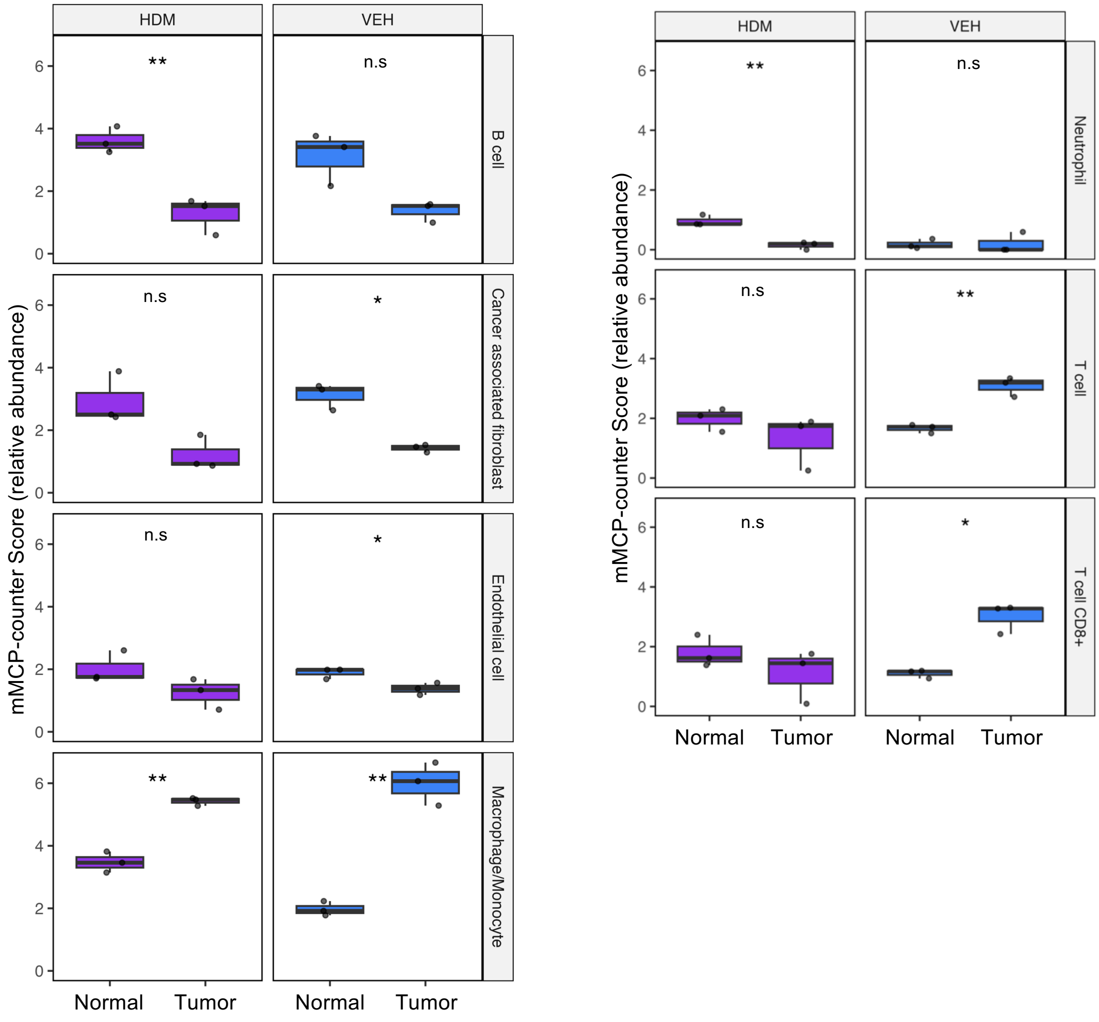

Figure S6. HDM Exposure Differentially Modulates Immune Cell Types in Tumors and Normal Adjacent Lung Tissues. Boxplots depict mMCP-counter scores for the indicated cell populations in tumors and normal lung tissues from mice treated with either HDM (purple) or VEH (blue). Boxes show the median (center line) and interquartile range (IQR); whiskers extend to 1.5× the IQR; points are individual samples (HDM, n = 3; VEH, n = 3). *p*-values were computed using a two-sided Welch's *t*-test; n.s: non-significant, * $p < 0.05$, ** $p < 0.01$. Benjamini-Hochberg false discovery rate (FDR) was controlled at 10% (q ≤ 0.1) across cell types within each exposure, and panels were selected based on this FDR criterion.

bioRxiv preprint doi: https://doi.org/10.1101/2025.09.24.678347; this version posted September 29, 2025. The copyright holder for this preprint (which was not certified by peer review) is the author/funder, who has granted bioRxiv a license to display the preprint in perpetuity. It is made available under aCC-BY 4.0 International license.

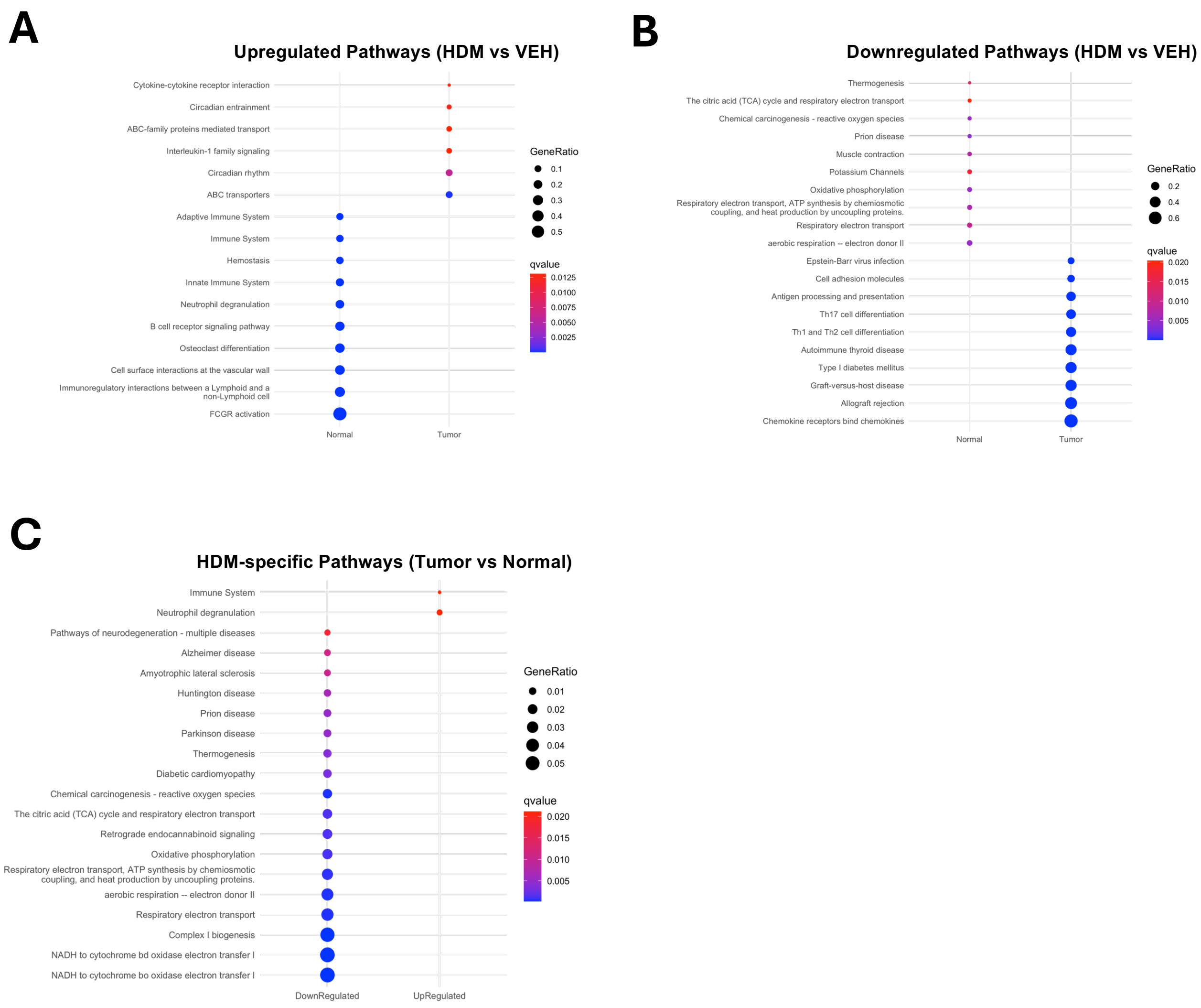

Figure S7. HDM Exposure Alters Signaling Pathways in Tumors and Normal Adjacent Lung Tissues. Pathway enrichment analysis of DEGs identified in tumor and normal adjacent lung tissues based on KEGG, Reactome, and MouseCyc pathway databases. The dot plots display all pathways (uncurated) that are either **A)** upregulated or **B)** downregulated in normal lung tissues or tumors following HDM exposure compared to VEH. **C)** Common pathways modulated by HDM in both normal lung tissues and tumors. Dot size represents the number of DEGs associated with each pathway, while dot color indicates statistical significance (q-value, calculated using the Benjamini-Hochberg false discovery rate).

bioRxiv preprint doi: https://doi.org/10.1101/2025.09.24.678347; this version posted September 29, 2025. The copyright holder for this preprint (which was not certified by peer review) is the author/funder, who has granted bioRxiv a license to display the preprint in perpetuity. It is made available under aCC-BY 4.0 International license.

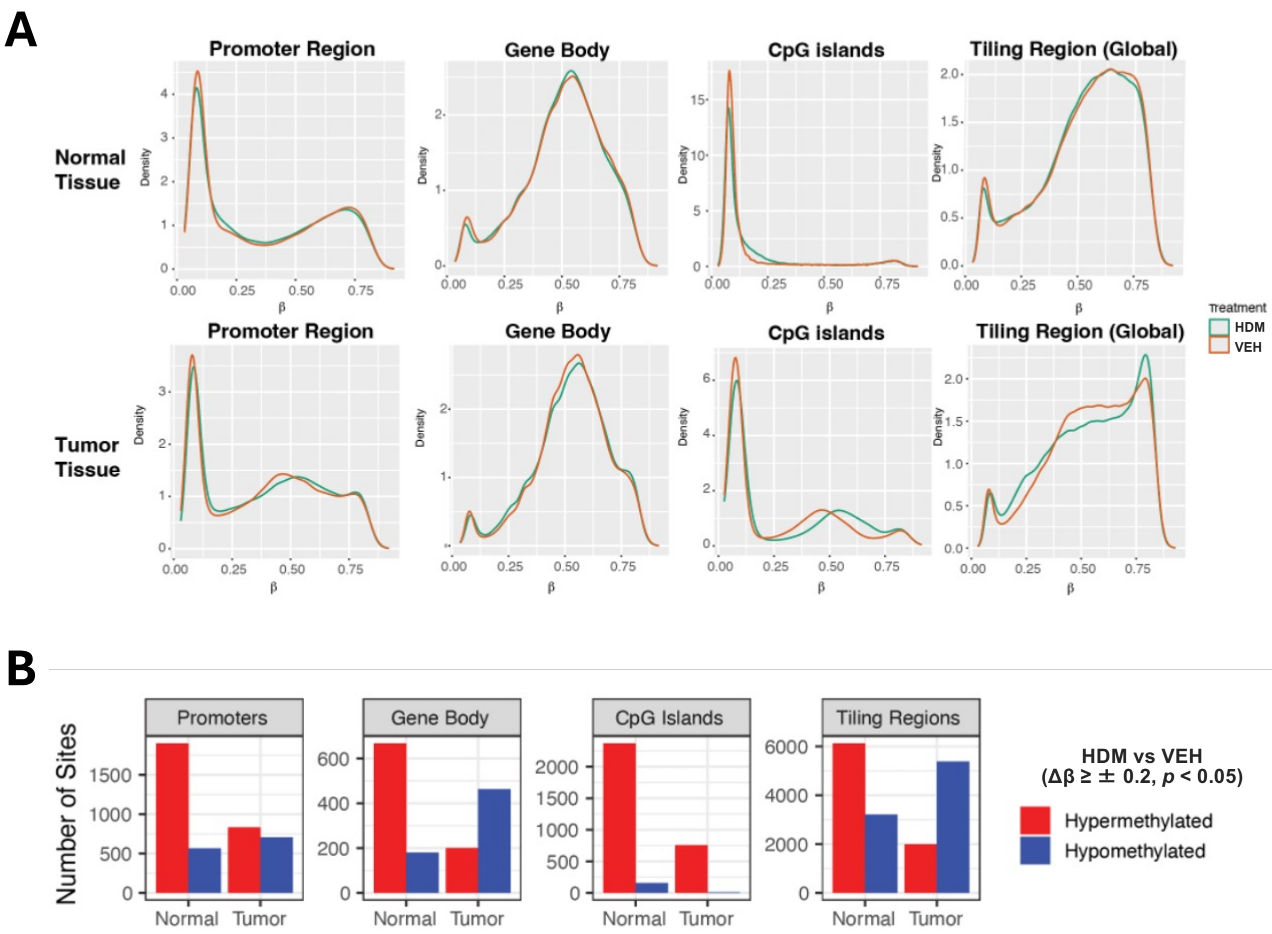

Figure S8. DNA methylation profiles of tumors and normal adjacent tissues exposed to HDM or VEH. A) β-value density plots. Kernel density curves showing the distribution of CpG β-values (0-1) across promoters, gene bodies, CpG islands, and genome-wide tiling regions for normal lung tissue (top row) and tumors (bottom row) under HDM (purple) and VEH (blue) conditions. **B)** Counts of differentially methylated sites (DMSs). Bar charts show the number of hypermethylated (red) and hypomethylated (blue) CpGs in each feature/tissue, defined as $|\Delta\beta| \geq 0.20$ and nominal $p < 0.05$ ($\Delta\beta$ = mean[HDM] − mean[VEH]).
